# Scoping review of dual-task interference in individuals with intellectual disability

**DOI:** 10.3389/fpsyg.2023.1223288

**Published:** 2023-08-24

**Authors:** Roi Charles Pineda, Ralf Th Krampe, Yves Vanlandewijck, Debbie Van Biesen

**Affiliations:** ^1^Department of Rehabilitation Sciences, KU Leuven, Leuven, Belgium; ^2^Brain and Cognition Group, Faculty of Psychology and Educational Sciences, KU Leuven, Leuven, Belgium; ^3^Department of Physiology, Swedish School of Sport and Health Sciences, Stockholm, Sweden; ^4^Virtus Academy, Virtus World Intellectual Impairment Sport, Sheffield, United Kingdom

**Keywords:** multitasking, executive function, Down syndrome, William syndrome, gait and balance, intellectual disability

## Abstract

Dual-task paradigms can provide insights on the structures and mechanisms underlying information processing and hold diagnostic, prognostic, and rehabilitative value for populations with cognitive deficits such as in individuals with intellectual disability (ID). In this paradigm, two tasks are performed separately (single-task context) and concurrently (dual-task context). The change in performance from single- to dual-task context represents dual-task interference. Findings from dual-task studies have been largely inconsistent on whether individuals with ID present with dual-task-specific deficits. The current review aimed to map the published literature on dual-task methods and pattern of dual-task interference in individuals with ID. A scoping review based on Arksey and O’Malley’s five-stage methodological framework was performed. Seventeen electronic databases and registries were searched to identify relevant studies, including gray literature. Charted data from included studies were analyzed quantitatively and qualitatively. PRISMA guidelines informed the reporting of this review. Twenty-two studies involving 1,102 participants (656 with ID and 446 without ID) met the review’s inclusion criteria. Participants in the included studies were heterogeneous in sex, age (range 3–59 years), etiology and ID severity. Included studies characterized their ID-sample in different ways, most commonly using intelligence quotient (IQ) scores. Other measures of intellectual function (e.g., mental age, ID severity, verbal and/or visuospatial ability scores) were also used, either solely or in combination with IQ. Methods of dual-task testing varied across studies, particularly in relation to dual-task combinations, equation of single-task performance between groups, measurement and reporting of dual-task performance for each single-task, and task priority instructions. Thematic content of the included studies were: (1) structural interference to dual-tasking; (2) etiology-based differences in dual-tasking; (3) gait and balance dual-task performance; (4) testing executive function using dual-task paradigms; and (5) training effect on dual-task performance. Although the evidence consistently supported the intact dual-tasking ability of individuals with ID, the pattern of dual-task interference was inconsistent. Likewise, the evidence was inconclusive regarding dual-task deficit specific to individuals with ID because of heterogeneity in dual-task study designs among included studies.

## Introduction

Dual-tasking is so extensively ingrained in everyday life that one may be simultaneously engaged in two tasks without much thought. However, dual-task failures like losing track of oral conversations while listening to the news or, more catastrophically, crashing one’s car while using a mobile device foreground the cognitively demanding nature of dual-tasking. These examples illustrate performance deterioration due to concurrent execution of two tasks. Researchers have capitalized on the phenomenon of dual-tasking related performance decrement to examine the structure and mechanisms underlying human information processing, particularly in conjunction with the effects of aging, pathology and/or practice. Moreover, dual-tasking has been argued to hold diagnostic, prognostic, and rehabilitative value for populations with cognitive deficits (Saccani et al., 2022). The current review focuses on dual-task performance in individuals with intellectual disability (ID), a developmental disorder characterized by impaired intellectual function and adaptive behavior (Schalock et al., 2021).

The determination of intellectual impairment is defined as a full-scale intelligence quotient (IQ) score of ≤70 (± 5 margin of error; World Health Organization, 2019; Schalock et al., 2021). Despite this straightforward diagnostic definition, every individual with ID exhibits varying profiles of strengths and weaknesses in cognitive abilities. Several factors such as etiological diversity (genetic, environmental, or idiopathic), presence of comorbidities (e.g., autism, epilepsy, attention-deficit hyperactivity disorder, etc.), timing of diagnosis (early versus late) and degree of available supports contribute to its heterogeneous clinical presentation (American Psychiatric Association, 2022). Level of ID severity was previously defined based on IQ score ranges: mild (IQ 50–55 to 70), moderate (IQ 35–40 to 50–55), severe (IQ 20–25 to 35–40), and profound (IQ < 20–25; American Psychiatric Association, 1994). However, current classification systems for ID severity have placed greater emphasis on functional skills and level of support needed rather than just IQ score (World Health Organization, 2019; Schalock et al., 2021; American Psychiatric Association, 2022). Nonetheless, owing to its simplicity, many studies continue to use IQ scores to define ID severity.

Dual-task interference is experimentally assessed using the dual-task paradigm. In this paradigm, two tasks are performed singly (single-task context) and simultaneously (dual-task context). The change in performance from single- to dual-task represents dual-task interference (Wickens et al., 1983; Koch et al., 2018). While its exact cause is still unclear, several models have been proposed to explain the neuromechanisms behind dual-task interference. The *resource model* assumes that dual-tasking splits resources between concurrent tasks and the resulting interference is caused by the two tasks competing for an individual’s finite resource (Norman and Bobrow, 1975; Pashler, 1994). With adequate resource, dual-tasking proceeds without compromising performance on either task; otherwise, performance decrement in one or both tasks can be expected. One point of divergence between researchers is the singularity or plurality of the resource pool. Within the model’s framework, resource is the mental facility that drives information processing (Norman and Bobrow, 1975) and has also been conceptualized in terms of attention (i.e., divided attention) (Posner and Boies, 1971), mental effort (Kahneman, 1973), processing speed (Birren, 1974; Verhaeghen et al., 2003) and working memory capacity (Baddeley and Hitch, 1974), among others. Some researchers (Kahneman, 1973) have subscribed to the idea that a general resource (i.e., unitary resource) adequately explains dual-task interference. Alternatively, others (Navon and Gopher, 1979; Wickens et al., 1983) have suggested the existence of multiple resources (e.g., verbal, spatial, etc.) and contended that the success or failure of dual-task performance depends on the amount of overlap in the type of resource required by the component tasks.

The allocation of (general or specialized) resource between competing tasks is under some level of an individual’s control. The degree of control can be influenced by a number of factors related to the task (e.g., task emphasis instruction, task difficulty) or person (e.g., strategic bias, task mastery) (Pashler, 1994; Schumacher et al., 1999; Li et al., 2005; Yogev-Seligmann et al., 2012). This top-down control of resource allocation to multiple tasks lends dual-task paradigm as a method to assess executive functions (Della Sala et al., 1995; Baddeley et al., 1997; Miyake et al., 2000). Also called executive or cognitive control, executive functions refer to a number of high-order cognitive processes that regulate goal-directed behaviors, particularly in the face of novelty or change (Diamond, 2013; Friedman and Miyake, 2017). Researchers postulated that dual-task performance involves not only task-specific cognitive processes, but also other additional processes like *task coordination* (Kramer et al., 1995; Strobach and Torsten, 2017). It also remains unknown to what extent dual-task performance depends on unspecific cognitive processes. Because these other processes compete for the same limited resource, resource required for dual-task performance is understood to be greater than the sum of the two tasks’ processing demands.

Dual-tasking demonstrates the significant contribution cognition plays in the performance of several important daily living tasks. Performance of over-practiced and seemingly automatized tasks such as standing balance and walking (Woollacott and Shumway-Cook, 2002; Li et al., 2005; Al-Yahya et al., 2011; Boisgontier et al., 2013), listening (Gagné et al., 2017), and talking (Lee et al., 2017; Fournet et al., 2021) have shown susceptibility to dual-task interference. This point is further emphasized by greater vulnerabilities to dual-task interference reported in conditions with reduced cognitive capacities, including age-related cognitive decline in older adults (Verhaeghen et al., 2003; Boisgontier et al., 2013) and neurocognitive disorders like Parkinson (Raffegeau et al., 2019) and Alzheimer (Della Sala et al., 1995; Logie et al., 2004; Rapp et al., 2006). Similarly, individuals with ID can be expected to have worse dual-task performance and higher dual-task interferences relative to individuals with normal intelligence. This expectation is motivated by at least two reasons. First, impaired intellectual functioning translates to reduced resource. This is corroborated by the strong association between intelligence and mental resources such as attention (Schweizer et al., 2005; Cowan et al., 2006), working memory (Conway et al., 2003; Conway and Kovacs, 2013) and processing speed (Kail, 2000; Sheppard and Vernon, 2008). Following the prediction of the resource model, a smaller resource pool would restrict what is available for single- and dual-task performance and increase vulnerability to dual-task interference. Second, there is evidence supporting impaired executive function in individuals with ID (Spaniol and Danielsson, 2022; Van Biesen et al., 2023), which could hinder efficient allocation of resource and coordination of component tasks during dual-task performance.

The critical question, however, is whether individuals with ID experience challenges during dual-tasking over and above what can be accounted for by their reduced performance in the component single-tasks (i.e., dual-task-specific deficit). Several studies (Mohan et al., 2001; Van Biesen et al., 2018; Kachouri et al., 2020) reported poorer dual-task performance and larger dual-task interference in participants with ID compared to participants without ID. However, there are also findings demonstrating otherwise (Van der Molen et al., 2007; Oka and Miura, 2008). A number of methodological factors may account for these conflicting results. One consideration is related to the heterogeneity of etiology and clinical presentation of ID. Genetic syndromes typically display distinct cognitive profiles while non-specific ID presents with a diffuse pattern of strengths and weakness. Comparing Down syndrome (DS) with William syndrome (WS), for instance, the former has been shown to perform better in verbal than in visuospatial tasks while the reverse is true for the latter (Fidler et al., 2016). In developmental disabilities studies, researchers typically recruit controls who match participants with ID by chronological age (CA) or mental age (MA) and either approach has their own advantages or disadvantages (Russo et al., 2021). A study’s choice of comparison group and criteria for matching contextualizes and constrains. How its result can be interpreted and compared to other dual-task studies. Other factors relate to the experimental dual-task procedure itself. Equating single-task performance between groups is an important consideration. This is because group differences in dual-tasking can be accounted for by differential baseline performance on component single-tasks rather than group differences in dual-tasking ability (Anderson et al., 2011). It is also critical that researchers investigate dual-task interferences on both tasks. Considering the possibility of mutual task interference and task prioritization (Schaefer, 2014; McIsaac et al., 2015), dual-task findings from only one of the tasks can be misleading. Researchers’ task priority instructions to study participants can influence dual-task performance. As individuals exert some degree of control over their resource allocation when dual-tasking (Li et al., 2005; Fraizer and Mitra, 2008), test instructions can bias allocation priority.

In light of conflicting findings, a review can help consolidate our understanding of the effect of ID on dual-task interference and advance the application of the paradigm in the ID population. However, to the best of our knowledge, no reviews have summarized dual-task studies on individuals with ID. We therefore aimed to survey published research on the topic, emphasizing on describing the methods used to measure dual-task performance and the pattern of dual-task interference in individuals ID. Considering the heterogeneity in the population of interest with regards to age, ID severity, etiology and comorbidity, and varied configuration of tasks (and outcome measures) in dual-task paradigms, we implemented a scoping review. Unlike systematic reviews which focus on well-defined research questions, scoping reviews answer broader questions and allow the inclusion of more diverse research designs (Arksey and O’Malley, 2005; Levac et al., 2010; Munn et al., 2018). A scoping review is particularly helpful in mapping the breadth of available literature in previously unreviewed research areas, finding gaps in extant literature, and determining the need for and feasibility of a full systematic review (Mays et al., 2001; Arksey and O’Malley, 2005; Daudt et al., 2013).

## Methods

For the review protocol, we adopted the methodological framework described by Arksey and O’Malley (2005) and enhanced by succeeding authors (Levac et al., 2010; Daudt et al., 2013). The framework involves a five-stage process: (1) identify the research question, (2) identify relevant studies, (3) select studies, (4) chart data, and (5) collate, summarize and report results. Reporting in this review was guided by the PRISMA extension for scoping reviews (Supplementary Table 1; Tricco et al., 2018).

### Identifying the research question

We asked the broad question: *how does ID impact on dual-task interference?* Specifically, we focused on describing the determinants of dual-task interference related to the characteristics of study participants, task combinations used, and testing procedures. For this review, we defined dual-task performance as the concurrent performance of two tasks that each have distinct goals and can be performed and measured independent from the other while dual-task interference is the resulting change in performance from single- to dual-task contexts (McIsaac et al., 2015). To distinguish it from the related task-switching paradigm, the task processing for the two tasks should temporally overlap, as in the simultaneous presentation of task stimuli (Koch et al., 2018). We adopted the definition of ID as a disability characterized by significant impairments of intellectual functioning and adaptive behavior which have been present before 22 years of age (Schalock et al., 2021). These operational definitions informed the scope of the study considering the broad nature of the research question (Levac et al., 2010).

### Identifying relevant studies

In consultation with reference librarians, we compiled search terms from a combination of free text and database-specific controlled vocabularies like MeSH and Emtree based on the two key concepts of this review—dual-task and ID—and developed our search string. We incorporated related terms to dual-tasking such as multitasking, task interference, concurrent task, divided attention, task coordination, and executive function. For ID, we included learning disability, which is the preferred term in the United Kingdom (National Institute for Health and Care Excellence, 2015), and other terms like intellectually challenged or mental retardation, as well as DS and Fragile X syndrome, which are the most common genetic cause and most common inherited form of ID, respectively (McDermott et al., 1995). For comprehensiveness, we searched several electronic databases, as well study registries to cover the gray literature. The exact search strings used for each database/register can be found in Supplementary Table 2. Lastly, we manually identified additional articles by executing a backward and forward citation search of the included studies using Scopus and Web of Science.

### Study selection: criteria for inclusion and exclusion of articles

Bibliographic information of all articles identified in the search was imported to EndNote X9 (Clarivate, Philadelphia, United States). Following the procedure outlined by Bramer et al. (2016), we used EndNote’s de-duplication feature to eliminate identical records. RCP and DVB independently screened the records on Rayyan, a web-based application for literature reviews (Ouzzani et al., 2016), in two phases. Each record’s title and abstract was first screened against our inclusion criteria. Records that met our inclusion criteria or were difficult to judge based on the title and abstract alone were moved to the next phase of screening. Full-text articles of remaining records were retrieved for the second screening to examine eligibility. Disagreements between reviewers at either phase of screening were discussed. Inclusion criteria include: (1) primary quantitative research; (2) human participants with ID regardless of etiology; and (3) performance in single-task and dual-task context is compared, with reported data on single- and dual-task performance in at least one of the experimental tasks. The third criteria was relevant to exclude studies where it would be impossible to determine dual-task interference (e.g., studies with no single-task performance measure) or to isolate the effect of one component single-task on the other during dual-task performance (e.g., studies where outcomes of dual-task performance for each single-task cannot be separated). In the first phase of screening, we also excluded articles whose title and abstract were not in English. Full-texts in foreign languages that passed the first screening were translated to English for the second screening and subsequent charting. No limits were applied on publication date. Studies from gray literature, such as conference proceedings and graduate dissertations, were included.

### Charting the data

The same two researchers charted the data independently. To determine which information to extract from the included articles, a charting form was iteratively developed whereby the initial charting form was continually updated as relevant information related to the research question emerge from reading, re-reading, and charting the different articles. The two researchers met after charting a set of ten articles and discussed adaptations to the form, which were then retrospectively applied to any articles charted previously. For all articles, extracted information included: (1) bibliographic information; (2) purpose of using a dual-task paradigm; (3) participant characteristics like demographics, ID etiology, and measure of intellectual functioning; (4) dual-task testing details, including experimental tasks, performance measure, instruction on task prioritization, and between-group difference at the level of single-task; and (5) key results.

### Collating, summarizing, and reporting the results

Implementation of the last stage followed the three steps outlined by Levac et al. (2010). First and second steps were the quantitative and qualitative analysis of charted data, respectively. With a descriptive quantitative summary of the extent, nature and distribution of included articles, not only dominant characteristics of published reports but also significant gaps in knowledge on the topic can be uncovered (Arksey and O’Malley, 2005). Meanwhile, a qualitative thematic analysis (Braun and Clarke, 2006, 2012), which we implemented in Atlas.ti 7 (Scientific Software Development GmbH, Berlin, Germany), guided the synthesis of textual information from charted data and full-text articles. The process of coding and creating themes drew on dual-task theories. Lastly, we reported results of both analyses and identified broader implications, limitations and recommendations for future research and practice.

## Results

**Figure 1:**
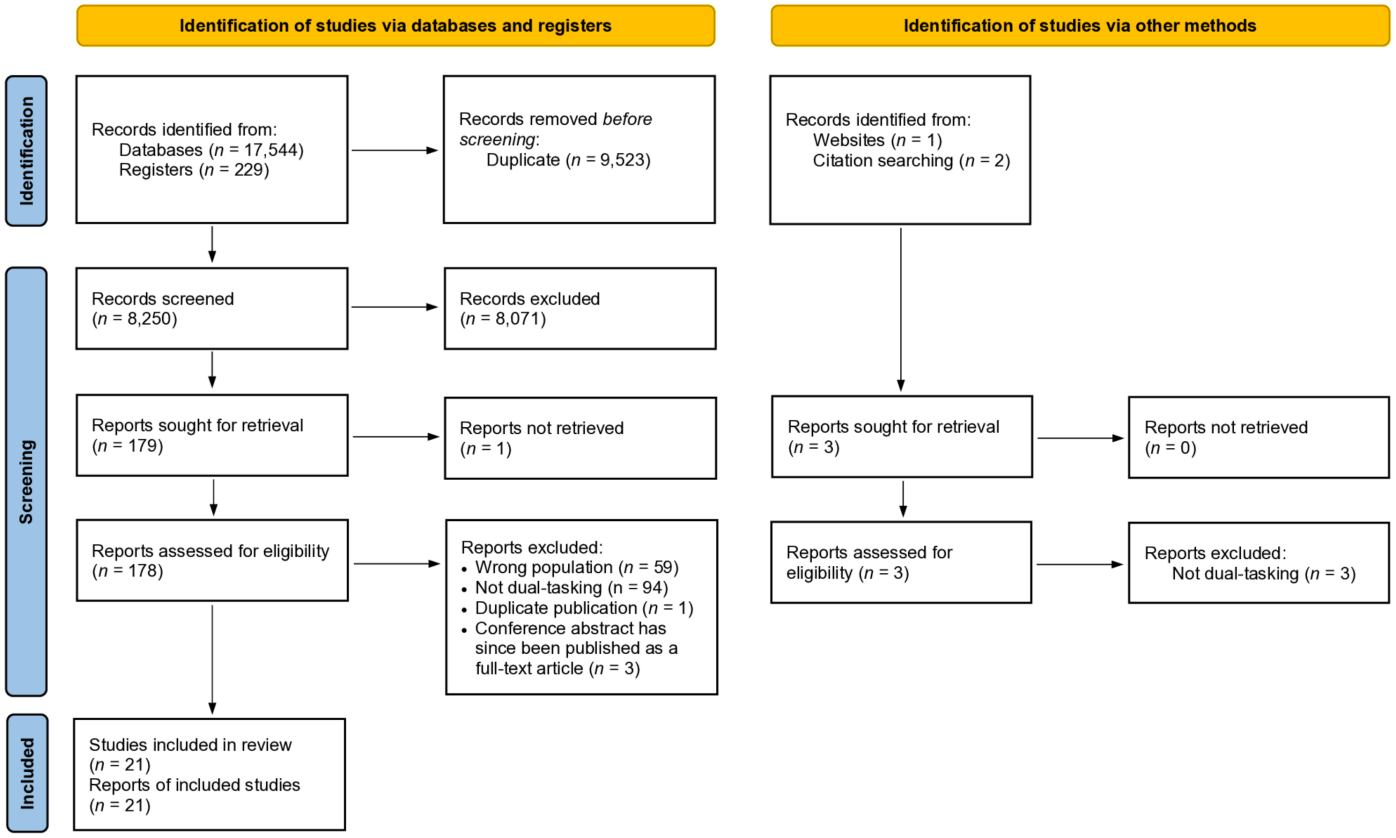
PRISMA flow diagram of the first iteration of study selection carried out in January 2021.

Our initial database and study register search in January 2021 yielded 8,250 deduplicated records. Following the title and abstract screening, 8,071 records were excluded. All remaining records underwent full text screening, except for one article (Lanfranchi et al., 2003) which we could not retrieve even after contacting the corresponding author. We further excluded 157 records, leaving us with 21 articles after the first iteration of the study selection process (Figure 1). We updated the search in January 2022 and found one record that met our inclusion criteria (Supplementary Figure 1). Because we did not find any additional records from citation search, this scoping review included 22 studies.

### General description of included studies

Study participants’ characteristics are presented in Table 1. In characterizing the ID sample, IQ was the most commonly used metric for intellectual function (8/22). Other studies used other measures, such as mental age (3/22), ID severity (e.g., mild ID; 4/22), verbal and/or visuospatial abilities (3/22), either solely or in combination with IQ. Information on intellectual functioning was unavailable in four studies but their participants had confirmed genetic syndromes that commonly present with ID. In articles where ID severity could be identified (19/22), study participants were all classified as mild/moderate ID, except for one study that recruited participants with severe ID (Parlow et al., 1996). The etiology of the primary ID sample was genetic in 12 studies and non-specific (i.e., ID with no known genetic cause) in four studies. DS was the most commonly examined ID subgroup (10/12). Individuals with ID of mixed etiologies were the participants in two studies. Four studies did not specify the etiology of their ID samples. For age groups, 14 studies (64%) involved adults with ID while eight studies were of children/adolescents with ID. Except for one study that recruited only males (Piccirilli et al., 1991), ID groups in the majority of studies (16/22) are composed of both males and females. Five studies, however, omitted reporting information about the participants’ sex.

**Table 1:** Participant demographics of included studies.

**Reference**	***N***	**Participants (matched by)**	**Sex, M/F**	**Mean age, in years**	**Mean intellectual functioning**
Abbruzzese et al. (2016)	14	Cri du Chat	2/12	10.3 (SD = 5.7, range = 3–20)	NR
	14	CT (CA)	4/10	10.1 (SD = 5.4, range = 3–20)	NR
Elliott et al. (1987)	12	DS	6/6	(range = 16–33)	MA = 6.5 yrs.
	12	CT (CA)	6/6	(range = 18–35)	NR
Hocking et al. (2013)	16	WS	9/7	24.2 (SD = 6.9)	MA = 10.4 yrs. (SD = 2.2); BPVS = 132.9 (SD = 15.2); RPM = 20.6 (SD = 6.5)
	16	CT (verbal ability)	8/8	9.0 (SD = 1.6)	MA = 9.8 yrs. (SD = 2.3); BPVS = 123.6 (SD = 17.7); RPM = 29.8 (SD = 3.8)
	16	CT (spatial ability)	10/6	8.1 (SD = 1.4)	MA = NR; BPVS = NR; RPM = 24.8 (SD = 3.7)
Hocking et al. (2014)	18	WS	7/11	26.2 (SD = 7.3)	BPVS = 127.8 (SD = 18.3); RPM = 19.9 (SD = 6.6)
	17	DS	8/9	24.8 (SD = 3.0)	BPVS = 92.2 (SD = 29.4); RPM = 18.0 (SD = 4.3)
	17	CT (CA)	11/6	23.2 (SD = 6.1)	NR
Horvat et al. (2013)	12	DS	NR	22.8 (SD = 3.1)	NR
	12	CT (CA, sex)	NR	22.5 (SD = 3.2)	NR
Kachouri et al. (2020)	15	NSID	NR	8.6 (SD = 1.4)	IQ = 61.8 (SD = 2.0)
	15	CT (CA)	NR	8.9 (SD = 1.7)	IQ = 88.2 (SD = 2.9)
Kittler et al. (2008)	53	DS	24/29	44.7 (SD = 7.2)	IQ = 53.4 (SD = 11.9)
	10	WS	3/7	47.7 (SD = 16.6)	IQ = 61.6 (SD = 6.1)
	39	NSID	13/26	53.7 (SD = 10.2)	IQ = 56.4 (SD = 10.2)
Lee et al. (2010)	10	ID, unspecified	5/5	30.1 (SD = 6.4)	mild ID (*n* = 3); moderate ID (*n* = 7)
	10	CT	0/10	22.3 (SD = 0.5)	NR
Medenica et al. (2010)	37	ID, unspecified	21/16	12.1 (SD = 2.0; range = 7–15)	IQ = 60.2 (range = 49–72)
Merrill and Peacock (1994)	24	ID, unspecified	NR	17.2 (SD = 1.7)	IQ = 62.3 (SD = 6.8)
	24	CT	NR	18.2 (SD = 0.4)	NR
Mohan et al. (2001)	20	ID, mixed	12/8	17.5 (SD = 3.4)	IQ range = 55–76
	20	CT (CA)	12/8	17.1 (SD = 2.5)	IQ range = 90–104
Oka and Miura (2008)	16	ID, unspecified	8/8	21.1 (SD = 3.0, range = 18–28)	mild ID (*n* = 11); moderate ID (*n* = 5)
	16	CT (CA)	8/8	23.8 (SD = 2.3, range = 20–28)	NR
Oppewal and Hilgenkamp (2019)	31	ID, mixed	24/7	42.8 (SD = 16.7)	mild ID (*n* = 15); moderate ID (*n* = 16)
Parlow et al. (1996)	8	DS	3/5	42.0 (SD = 6.1)	IQ = 29.9 (SD = 12.2)
	8	ID, non-DS	NR	40.1 (SD = 5.4)	IQ = 31.4 (SD = 11.6)
Pena et al. (2019)	21	DS	11/10	10.3 (SD = 2.3)	NR
	26	CT (CA)	9/17	10.2 (SD = 2.4)	NR
Piccirilli et al. (1991)	10	DS	10/0	28.8 (range = 15–36)	RPM = 19.4 (range = 12–24)
	10	ID, non-DS	10/0	24.4 (range = 16–35)	RPM = 19.1 (range = 12–25)
	10	CT (MA)	10/0	8.2 (range = 7–9)	RPM = 20.0 (range = 15–24)
Pineda et al. (2022)	29	NSID	24/5	25.4 (SD = 6.0)	IQ = 60.7 (SD = 7.2)
	29	CT (CA, sex, sports, training)	24/5	24.3 (SD = 6.2)	IQ = 112.9 (SD = 14.1)
Rao et al. (2017) ^§^	8	DS	NR	All participants: range = 9–17	NR
	8	ID, non-DS	NR		NR
	8	CT (CA, sex)	NR		NR
Shaw (1998)	13	DS	NR	30.6 (SD = 6.3)	MA = 6.09 yrs. (SD = 2.2)
	16	ID, non-DS	NR	38.1 (SD = 8.1)	MA = 7.1 yrs. (SD = 2.4)
	24	CT (MA)	NR	NR	MA = 7.8 yrs. (SD = 2.5)
	24	CT (CA)^†^	NR	32.0 (SD = 8.5)	NR
Van Biesen et al. (2018)	103	NSID	33/70	24.4 (SD = 5.8)	IQ = 60.6 (SD = 9.2)
	103	CT (CA, sex, sport, training)	33/70	22.0 (SD = 2.4)	NR
Van der Molen et al. (2007)	50	NSID	38/12	15.3 (SD = 1.0)	MA = 10.7 yrs. (SD = 23.5)
	25	CT (MA)	17/8	11.0 (SD = 1.1)	MA = 11.0 yrs. (SD = 17.6)
	25	CT (CA)	17/8	15.1 (SD = 0.8)	MA = 15.0 yrs. (SD = 19.8)
Van Pelt et al. (2020)	28	DS, 6 with dementia	15/13	36.6 (SD = 7.0, range = 25–59)	borderline/mild ID (*n* = 16); moderate ID (*n* = 12)

§ Charted only the final analysis, which describes a subset of participants who were able to perform all experimental conditions.

† Author declared that they recruited chronological age-matched (CA) controls (CT) although final sample showed significant age difference between groups.

MA, mental age; ID, intellectual disability; NSID, non-specific intellectual disability; WS, William syndrome; DS, Down syndrome; IQ = intelligence quotient; BPVS, British Picture Vocabulary Scale; RPM, Raven’s Progressive Matrices; NR, not reported.

Included articles comprised mostly of between-group comparative studies: 13 studies were between participants with ID and non-ID controls, two studies compared exclusively between ID subgroups (e.g., DS versus WS or DS versus non-specific ID), and four studies included both ID subgroups and controls. The remaining three articles were single-group studies. All studies employed convenience or criterion sampling. Sample size per group ranged from eight (Parlow et al., 1996) to 103 (Van Biesen et al., 2018) but half of the studies have ≤18 participants per group. Only one study (Pineda et al., 2022) reported power calculation to justify its sample size. One study attributed their small sample size to the difficulty of recruiting participants with Cri du Chat syndrome (CdC), a rare genetic condition (Abbruzzese et al., 2016). Of the 17 studies with control groups, 11 used CA-matched controls, two used MA-matched controls, two recruited two control groups—one MA-matched and another CA-matched—and two had controls who were not matched with participants with ID in either CA or MA.

Table 2 outlines the dual-task procedures implemented across the included studies. Task priority instructions were explicitly reported in nine studies, eight of which had instructions to perform both tasks equally well and one study emphasized performance of one task over the other (Merrill and Peacock, 1994). As to the reporting of task performance, the majority of studies reported single- and dual- task performance for only one of the component tasks and only nine studies completely reported single- and dual-task performance on both component tasks. Equating single-task performance across groups was done in two of the 19 between-group comparative studies (Hocking et al., 2013, 2014) but there were another four studies wherein group difference in single-task performance was not significant despite not being methodologically controlled (Parlow et al., 1996; Kittler et al., 2008; Kachouri et al., 2020; Pineda et al., 2022). Dual-task interference was quantified as proportional dual-task interference in half of the included studies. Ten of these studies reported proportional dual-task interferences for each component task separately and one averaged the dual-task interferences from both component tasks to report a single metric for the combined dual-task interference (Van der Molen et al., 2007). Studies that did not report dual-task interference used ANOVA group x task-context interaction effects to test between-group differences in dual-task performance that is over and above between-group differences in single-task performance.

**TABLE 2:** Description of dual-task method of included studies, including task priority instruction, type of task, measure of task performance, direction of dual-task interference (DTI), and group difference in single-task (ST) performance and DTI.

Reference	Task priority	Task 1					Task 2				
		Task	Measure	DTI direction	Group difference		Task	Measure	DTI direction	Group difference	
					ST	DTI				ST	DTI
Abbruzzese et al. (2016)	NR	Walking (self-paced)	Step length and cadence	Task 2a: CdC: ↔ CT: ↓	CT > CdC	Task 2a: CT > CdC	a. Carrying pitcher	Spill or no spill (categorical)	NT	CdC > CT	NT
				Task 2b: CdC/CT: ↓		Task 2b: NS	b. Carrying tray	Not quantified		NT	NT
Elliott et al. (1987)	NR	a. Left finger tapping	# Taps	DS, CT: ↓	CT > DS	NS	Sound-shadowing	# errors	NR	NS	NR
			Tap variability	DS, CT: ↑	DS > CT	DS > CT					
		b. Right finger tapping	# Taps	DS, CT: ↓	CT > DS	NS					
			Tap variability	DS, CT: ↑	DS > CT	DS > CT					
Hocking et al. (2013)	Equal	Choice stepping reaction task	Reaction time	Task 2a/b: WS, CT_v_, CT_s_: ↑	WS > CT_v_ WS = CT_s_	Task 2a: WS > CT_s_; WS = CT_v_ Task 2b: WS > CT_v_, CT_s_	a. Semantic fluency^‡^ b. Inhibitory Go/No-Go	# Correct exemplars Accuracy	NS NT	NT NT	NT NT
			Movement time	Task 2a,b: WS, CT_v_, CT_s_: ↑	NS	Task 2a: WS > CT_v_, CT_s_ Task 2b: NS					
Hocking et al. (2014)	Equal	Walking (self-paced)	Velocity	Task 2a,b: WS, DS, CT: ↑	CT > DS; CT = WS	Task 2a: WS > CT Task 2b_1_: DS > CT	a. Semantic fluency^‡^ b. Digit span^‡^ 1. half 2. maximal	# Correct exemplars Accuracy	NS NT NT	NT NT NT	NT NT NT
			Step length	Task 2a,b: WS, DS, CT: ↑	CT > WS	Task 2a: WS > CT					
			Step time CoV	Task 2a,b: WS, DS, CT: ↑	DS > WS; DS > CT	Task 2a: WS > CT Task 2b_2_: DS > CT					
Horvat et al. (2013)	NR	Walking (self-paced)	Velocity	Task 2a,b: DS: ↔ Task 2c,d: DS: ↑	DS > CT	Tasks a–d: NR	a. Carrying tray and cup b. Carrying plate and cup	Not quantified Not quantified	NT NT	NT NT	NT NT
			Step length	Task 2a,c,d: DS: ↓ Task 2b: DS: ↔	DS > CT	Tasks a–d: NR	c. Buttoning shirt d. Talking on the phone	Not quantified Not quantified	NT NT	NT NT	NT NT
Kachouri et al. (2020)	Equal	a. Timed up and go	Completion time	Task 2a,b: ID, CT: ↑	ID > CT	Task 2a,b: ID > CT	a. Verbal fluency	# Incorrect exemplars	Task 1a,b: ID, CT: ↑	NS	Task 1a,b: NR
		b. Ten meter walking test	Completion time	Task 2a: ID, CT: ↑ Task 2b: ID: ↑ CT: ↔	ID > CT	Task 2a,b: ID > CT	b. Holding glass of water	Volume of spilled water	Task 1a,b: ID, CT: ↑	NS	Task 1a,b: NR
Kittler et al. (2008)	Equal	Peg placing	# Pegs placed in 30s	Task 2a: DS, WS ID: ↔	NS	Task 2a: NS	a. Repeat number	# Correct response	DS, WS, ID: ↓	NS	DS > WS, ID
				Task 2b: DS, WS, ID: ↓		Task 2b: DS > WS; DS > ID; WS = ID;	b. Next, number	# Correct response	DS, WS, ID: ↓	NS	DS > WS, ID
Lee et al. (2010)	NR	Memory for distance task	Absolute error	ID, CT: ↑	ID > CT	ID > CT	Mental arithmetic	Percent correct	NT	CT > ID	NT
			Variable error	ID, CT: ↑	ID > CT						
Medenica et al. (2010)	NR	Digit span^‡^	# Correct answer	ID: ↓	NA	NA	Visuomotor tracing task	# Circles connected	ID: ↓	NA	NA
Merrill and Peacock (1994)	Task 1	Card sorting:									
		a. Basic	Sorting time	ID, CT: ↑	ID > CT	NS	Auditory probe (press on foot pedal)	Cumulative response time	Task 1a,b: ID, CT: ↑	ID > CT	Task 1a,b: NR
		b. Super-ordinate	Sorting time	ID, CT: ↑	ID > CT	NS					
Mohan et al. (2001)	NR	a. Left finger tapping	# Taps	Task 2a,b: ID, CT: ↓	CT > ID	Task 2a,b: ID > CT	Story recall	NR	Task 1a,b: NT	NT	Task 1a,b: NT
		b. Right finger tapping	# Taps	Task 2a,b: ID, CT: ↓	CT > ID	Task 2a,b: ID > CT	Musical rhyme recall	NR	Task 1a,b: NT	NT	Task 1a,b: NT
Oka and Miura (2008)	Equal	Digit span^‡^	# Correct answer	ID, CT: ↓	CT > ID	NS	Visuomotor tracing task	# Crossed out boxes	ID, CT: ↔	CT > ID	NS
Oppewal and Hilgenkamp (2019)	NR	Walking (self-paced)	Gait measures: Velocity Step length Step time SD	ID: ↓ ID: ↓ ID: ↑	NA	NA	Speaking	Not quantified	NT	NA	NT
(Parlow et al., 1996)	NR	a. Left finger tapping	# Taps	Task 1a,b: DS, ID: ↓	NS	Task 1a,b: NS	a. Speaking	Not quantified	NT	NT	NT
		b. Right finger tapping	# Taps	Task 1a,b: DS, ID: ↓	NS	Task 1a,b: NS	b. Humming	Not quantified	NT	NT	NT
Pena et al., (2019)	NR	Sit-to-stand	COP measures:								
			Amplitude	Task 2a,b: ID: ↓ CT: ↔	NR	Task 2a,b: ID > CT	a. Carrying tray and cups	Not quantified	NT	NT	NT
			Velocity	Task 2a,b: ID: ↓ CT: ↔	NR	Task 2a,b: ID > CT	b. Holding cup	Not quantified	NT	NT	NT
Piccirilli et al. (1991)	Equal	a. Right finger tapping	# Taps in correct sequence	DS, ID, CT: ↓	CT > DS ID = DS	ID > DS, CT	Picture naming	# Syllables uttered	Task 1a,b: DS, ID, CT: ↓	CT > DS; DS > ID	Task 1a,b: DS > ID, CT
		b. Left finger tapping	# Taps in correct sequence	DS, ID, CT: ↓	CT > DS ID = DS	ID > DS, CT					
Pineda et al. (2022)	Equal	Standing on rocking board	COP measures:								
			Path length	ID: ↓ CT: ↔	NS	ID > CT	Visual recognition	Accuracy	ID: ↓	CT > ID	ID > CT
			Sample entropy	ID: ↔ CT: ↑	NS	CT > ID			CT: ↔		
Rao et al. (2017) ^§^	NR	Simple finger tapping reaction task	Reaction time	Task 2a,b: DS, ID, CT: ↔	DS, ID > CT	Task 2a,b: NS	a. Listening to music	Not quantified	NT	NT	NT
			Reaction force	Task 2a,b: DS, ID, CT: ↔	CT > DS, ID	Task 2a,b: NS	b. Stationary pedaling	Not quantified	NT	NT	NT
Shaw (1998)	NR	a. Right finger tapping	Tapping rate	Task 2a: DS, ID, CT_MA_: ↓ CT_CA_: ↔ Task 2b–d: DS, ID, CT_MA_, CT_CA_: ↓	CT_CA_ > DS, ID, CT_MA_	Task 2a: DS > ID, CT_MA_; ID, CT_MA_ > CT_CA_ Task 2b–d: DS, ID, CT_MA_ > CT_CA_	Perceptual discrimination:				
							a. Object matching b. Line matching Production: c. Speaking d. Humming	# Stimulus pairs viewed # Stimulus pairs viewed Not quantified Not quantified	NT NT NT NT	NT NT NT NT	NT NT NT NT
		b. Left finger tapping	Tapping rate	Task 2a–c: DS, ID, CT_MA_: ↓ CT_CA_: ↔ Task 2d: DS, ID, CT_MA_, CT_CA_: ↓	CT_CA_ > DS, ID, CT_MA_	Task 2a: DS, ID, CT_MA_ > CT_CA_; DS > CT_MA_ Task 2b: DS, ID, CT_MA_ > CT_CA_ Task 2c: DS, ID, CT_MA_ > CT_CA_; DS, CT_MA_ > ID Task 2d: DS, CT_MA_ > CT_CA_					
Van Biesen et al. (2018)	Equal	Flamingo balance test	Time standing on beam	ID, CT: ↓	CT > ID	ID > CT	Multiple object tracking	# Correct trials	ID, CT: ↓	CT > ID	ID > CT
Van der Molen et al. (2007)	NR	Digit span^‡^	# Correct answers	ID, CT: ↓	NR	NS	Visuomotor tracing task	# Circles connected	ID, CT: ↓	NR	NS
Van Pelt et al. (2020)	NR	Walking (self-paced)	Gait measures:								
			Velocity Step length	DS: ↓ DS: ↓	NA	NA	Counting	NR	NT	NA	NA

Arrow points to the direction of change in score on the outcome measure.

‡ Task difficulty was individually calibrated.

§ Charted only the final analysis, which describes a subset of participants who were able to perform all experimental conditions.

† Rather than reporting dual-task interference for each task, authors reported the average interference on both task.

NA, not applicable; NR, not reported; NS, not significant; NT, not tested; CT, controls; CT_v_, verbal ability-matched controls; CT_s_, spatial ability-matched controls; CT_CA_, chronological age-matched controls; CT_MA_, mental age-matched controls; CdC, Cri du Chat syndrome; ID, intellectual disability; DS, Down syndrome; WS, William syndrome; CoV, coefficient of variation; SD, standard deviation; COP, center-of-pressure.

### Thematic content of dual-task studies on individuals with ID

Thematic analysis of the included studies identified five themes. While we distinguish a theme from another, overlap between themes exist. Moreover, several studies extended to multiple themes. The themes were: (1) structural interference to dual-tasking; (2) etiology-based differences in dual-tasking; (3) gait or balance dual-task performance; (4) testing executive function using dual-task paradigms; and (5) training effect of dual-task performance. Table 3 presents the included studies and the themes they cover.

**Table 3:** Thematic coverage of included studies.

Reference	Structural interference to dual-tasking	Etiology-based differences in dual-tasking	Gait/balance dual-task performance	Testing executive function using dual-task paradigms	Trainability of dual-task performance
Abbruzzese et al. (2016)			✓		
Elliott et al. (1987)	✓				
Hocking et al. (2013)				✓	
Hocking et al. (2014)		✓	✓	✓	
Horvat et al. (2013)			✓		
Kachouri et al. (2020)			✓		
Kittler et al. (2008)		✓			
Lee et al. (2010)				✓	
Medenica et al. (2010)				✓	
Merrill and Peacock (1994)				✓	
Mohan et al. (2001)	✓				
Oka and Miura (2008)				✓	✓
Oppewal and Hilgenkamp (2019)			✓		
Parlow et al. (1996)	✓	✓			
Pena et al. (2019)			✓		
Piccirilli et al. (1991)	✓	✓			
Pineda et al. (2022)			✓		✓
Rao et al. (2017)		✓		✓	
Shaw (1998)	✓	✓			
Van Biesen et al. (2018)			✓	✓	✓
Van der Molen et al. (2007)				✓	
Van Pelt et al. (2020)			✓		

#### Theme 1: structural interference to dual-tasking

The first theme draws from the *functional cerebral space model* (Kinsbourne and Hicks, 1978), which postulates that two tasks mediated by the same cerebral hemisphere are more likely to result in dual-task interference than tasks subserved by separate hemispheres. Five of the earliest citations included in this review cover this theme (Elliott et al., 1987; Piccirilli et al., 1991; Parlow et al., 1996; Shaw, 1998; Mohan et al., 2001). These studies used the functional cerebral space model to test the hypothesis that DS is characterized by a reversal of cerebral dominance for language (i.e., right instead of left hemisphere). Except for a study with a mixed-etiology sample of adolescents with ID (Mohan et al., 2001), studies included in this theme examined adults with DS. All five studies paired a finger-tapping task (right and left hand to engage the left and right cerebral hemisphere, respectively) with a verbal task. A right-hemisphere controlled non-verbal task was also included in three studies (Parlow et al., 1996; Shaw, 1998; Mohan et al., 2001).

None of the studies’ findings supported the atypical cerebral dominance for language in Down syndrome. Furthermore, comparisons between individuals with DS and ID (non-DS) showed no differences in pattern of cerebral dominance (Piccirilli et al., 1991; Parlow et al., 1996; Shaw, 1998). Taken together, these studies found that participants with and without ID (both DS and non-DS) have comparable patterns of left and right hemisphere dominance for verbal and non-verbal task, respectively. Participants with ID, however, differed from CA-matched controls in the magnitude of dual-task interference, with the former demonstrating significantly lower single-task performance and larger performance decrements in finger tapping for the verbal and non-verbal concurrent tasks alike (Elliott et al., 1987; Shaw, 1998; Mohan et al., 2001). Comparing MA-matched controls with participants with ID, task difficulty influenced pattern of performance. In contrast to Shaw’s (1998) simple finger tapping task which participants with ID and MA-matched controls performed similarly in single- and dual-task conditions, Piccirilli et al. (1991) relatively more complex alternate tapping of the index and middle fingers distinguished the two groups better with MA-matched controls performing better in single-task contexts and incurring lower dual-task interferences relative to participants with ID.

#### Theme 2: etiology-based differences in dual-tasking

The theme, which is divided into two subthemes, highlights the differences in dual-task performance between known etiologies (i.e., causes) of ID. The first subtheme pertains to contrasting the dual-tasking ability between DS and WS. In particular, the subtheme includes two dual-task studies that examined whether differing cognitive profiles between DS and WS (i.e., the former is typified by relative strengths in visuospatial compared to verbal abilities and the reverse in the latter) would affect patterns of dual-task performance in adults with these syndromes. Kittler et al. (2008) and Hocking et al. (2014) had participants perform a motor task (peg placing and walking, respectively) and a concurrent verbal task with two levels of difficulty (repeat/next number and half/maximal digit span, respectively). Although no group differences were found in any of the single-task performance, only Kittler et al. found the expected larger dual-task interferences in DS relative to WS, which they observed in the verbal task performance regardless of difficulty level, as well as in the motor task performance but only when paired with the more difficult verbal task. Hocking et al. reported no difference in dual-task interference in the motor task between DS and WS but did find larger interference in DS relative to CA-matched controls. Nothing can be said about interferences in the verbal task because Hocking et al. only measured single- and dual-task performances in the walking task. Hocking et al. also included a concurrent semantic fluency task, which taps into visuospatial processing, and showed larger dual-task interferences in walking performance in WS relative to CA-matched controls, but not relative to DS.

The second subtheme concerns dual-task studies contrasting DS from non-DS etiologies of ID. Studies contributing to this subtheme involved a finger-tapping task and a verbal concurrent task out of consideration for the verbal-processing weakness noted in DS. Findings from three studies were in agreement that dual-task decrement in the finger-tapping performance of participants with DS were no different from participants with non-DS ID (Parlow et al., 1996; Shaw, 1998; Rao et al., 2017). Having reported single- and dual-task performance on both finger-tapping and verbal tasks, Piccirilli et al. (1991) showed the difference between the two groups lies in which task absorbs the dual-task interference. Dual-tasking resulted in diminished performance in the verbal task and finger-tapping task for participants with DS and non-DS ID, respectively.

#### Theme 3: gait or balance dual-task performance

Included in this theme are six dual-task gait studies and three dual-task balance studies. Four dual-task gait studies are comparative studies between participants with ID and CA-matched controls. In two studies, children (Kachouri et al., 2020) and young adults (Hocking et al., 2014) with ID, compared to controls, demonstrated larger dual-task interference (i.e., reduced gait velocity) when gait is combined with a cognitive task. Kachouri et al. also noted that dual-task interference in gait velocity was larger with a motor than a cognitive concurrent task. In Abbruzzese et al. (2016) study, children with ID, specifically CdC, showed similar or less dual-task gait interference with a concurrent motor task. The researchers attributed this finding to children with CdC’s lack of attention to the concurrent task and/or difficulty in modifying gait to accommodate the concurrent task. Horvat et al. (2013) reported that the dual-task effects of a concurrent motor task in young adults with ID are decreased gait efficiency and increased gait variability but their study did not analyze whether the magnitude of these effects is larger in participants with ID relative to controls. The other two dual-task gait studies are single-group studies that examined the value of dual-task gait interference for fall prediction (Oppewal and Hilgenkamp, 2019) and early detection of dementia (Van Pelt et al., 2020) in adults with ID. These studies found that dual-task interference on gait was associated to neither fall incidence nor dementia diagnosis but could not definitively dismiss its value because both studies lacked the sample size to address their primary research question. While effects did not reach significance, Oppewal and Hilgenkamp (2019) found medium effect sizes (*r* = 0.31–0.48) for the association between falls and gait parameters like base of support and stride time variability. Additionally, in a subgroup analysis based on ID severity, Van Pelt et al. (2020) identified a dual-task reduction in gait velocity in adults with mild ID while those with moderate ID demonstrated no dual-task gait interference.

Dual-task balance studies compared CA-matched controls with participants with ID. Two studies found greater dual-task balance instability for two different balance tasks: static one-legged standing (Van Biesen et al., 2018) and dynamic sit-to-stand balance task (Pena et al., 2019). Pineda et al. (2022) found the reverse pattern in a bipedal rocking board standing task whereby participants with ID improved stability when dual-tasking while controls showed no dual-task interferences. Based on sample entropies of center-of-pressure excursion, which is a measure of balance control automaticity, the researchers differentiated the dual-task balance strategy used by the two groups. Controls chose to leave their balance to automatic control in response to the increased challenge of dual-tasking while participants with ID maintained cognitive control over their balance. Such a strategy allowed participants with ID to maintain stability when dual-tasking albeit at the expense of the concurrent cognitive task. Performance in the concurrent task was only reported in two studies (Van Biesen et al., 2018; Pineda et al., 2022), both of which found larger dual-task decrement in the concurrent cognitive task (visual memory and object tracking task, respectively) for adults with ID compared to controls.

#### Theme 4: testing executive function using dual-task paradigms

Two cognitive models of executive function served as the theoretical basis for assessing dual-task performance in ID. Several studies adopted the *three-component working memory model* of executive function (Baddeley et al., 1997) and used verbal and/or visuospatial tasks to load on the two subsystems—*phonological loop* and *visuospatial sketchpad*, respectively—under the control of the *central executive*. Lee et al. (2010) used two visuospatial tasks, memory for distances and mental arithmetic, and found larger dual-task interference in accuracy of estimated distance in adults with ID. Three studies adopted the *pencil-and-paper task*, which combines verbal and visuospatial processing using digit span and visuomotor tracing tasks, respectively. Medenica et al. (2010) single-group study reported dual-task decrement in both verbal and visuospatial task performances for adults with ID. They further noted greater interference in the visuospatial task performance for those with the lowest IQ relative to the highest IQ scores. Oka and Miura (2008) found dual-task interference affected adults with ID in both tasks but the magnitude of interference in either task shown by the participants with ID was no different from CA-matched controls. Van der Molen et al. (2007) likewise found no group differences in dual-task interference, as measured by μ, a single dual-task interference score from the combined proportional dual-task interferences from each component tasks. In contrast to these studies, Hocking et al. (2013) paired a choice reaction time task with either an inhibitory Go/NoGo task or a semantic fluency task. These concurrent tasks tap into core executive functions under *unity/diversity model* of executive function (Miyake et al., 2000). Compared to MA-matched controls, adults with ID (specifically WS) had larger dual-task interference to stepping reaction time with either concurrent task. The dual-task effect of increased stepping reaction time correlated significantly with stepping accuracy suggestive of speed-accuracy trade-off for participants with ID but not controls. Pattern of dual-task performance in the concurrent executive function tasks were not reported.

Other studies have looked at dual-task performance at varying levels of required attentional control. One study by Rao et al. (2017) tested children with ID and CA-matched controls with a reaction time task concurrently performed with either a passive (listening to music) or an active (stationary pedaling) concurrent task. Both groups showed no dual-task interferences in reaction time with either type of concurrent task. The researchers speculated that neither concurrent task provided enough of a challenge to cause an interference to dual-task performance. However, the researchers noted that >40% of the original sample of children with ID could not perform both dual-task conditions. Examining attention allocation specifically, Merrill and Peacock (1994) had adults with ID and non-age-matched controls perform two types of card sorting task, basic or superordinate, with a concurrent auditory probe task. Although they found no group difference in dual-task interference in response time to auditory probes with the concurrent basic sorting task, controls showed larger interferences in superordinate card sorting speed. Together with the finding of larger decrease in sorting speed between basic and superordinate card sorting in adults with ID even when the instruction was to prioritize card sorting, the researchers interpreted this as failure of participants with ID to direct more attention to card sorting as task difficulty increased. Van Biesen et al. (2018) similarly reported reduced ability to allocate attention in participants with ID who, compared to controls, demonstrated larger error rates with increasing number of targets in a multiple object tracking task while doing a concurrent motor task. It was, however, not reported whether there were differences in motor task interferences across the difficulty levels of the multiple object tracking task.

#### Theme 5: training effect on dual-task performance

Studies in this theme cover expertise effect and practice effect in adults with ID. Two studies contrasted the cognitive-motor dual-task ability of elite athletes with ID against equally trained athletes without ID (CA-matched) and both found larger dual-task interferences on cognitive performance in the former (Van Biesen et al., 2018; Pineda et al., 2022). Neither study found advantage of athletes with ID in dual-task performance compared to athletes without ID. Doing repeated testing on multiple days, Oka and Miura (2008) examined practice effect on dual-task performance in participants with ID and CA-matched controls using the pencil-and-paper task described earlier. Single- and dual-task performances in the digit span and visuomotor tracing tasks equally improved for both groups between the first and final test session; however, μ scores indicated that participants with ID and controls showed comparable dual-task interferences and neither had any reduction in interferences due to practice.

## Discussion

This scoping review aimed to survey the empirical scholarship on dual-task interference in ID, particularly the methods of measuring dual-task performance and patterns of dual-task interference. We identified 22 articles satisfying the current review’s inclusion criteria and found large variations between studies in dual-task testing procedures related to single-task combinations, comparability of single-task performances between groups, measurement of dual-task interferences for each single-task, and task priority instructions. Although the majority of studies reported larger dual-task interferences in individuals with ID compared to CA-controls, evidence regarding dual-task-specific deficit in individuals with ID was inconclusive. This is due to inconsistencies in dual-task procedures across studies, which hindered comparability.

Researchers have long since identified methodological and interpretive issues with dual-task studies and possible solutions for these issues (Li et al., 2005; Fraizer and Mitra, 2008; McIsaac et al., 2015; Plummer and Eskes, 2015). Given the sizeable number of included studies that did not report dual-task interferences on all single-tasks, equate single-task performance between groups, and/or report task priority instruction, dual-task studies on individuals with ID have been inconsistent about addressing these issues. This casts doubts on the validity of conclusions made and prevents the synthesis of dual-task findings in ID. McIsaac et al. (2015) emphasized the value of examining performance trade-off on one task over another by measuring dual-task performance on all single-tasks. Looking at dual-task interference on only one of the single-tasks can lead to misleading conclusions, as when the absence of dual-task interference on the measured task is used as evidence for excellent dual-task ability. Unbeknownst to the researchers, such finding may have been afforded by a heavy dual-task interference on the unmeasured concurrent task (i.e., prioritization). Furthermore, the inconsistent reporting of task priority instruction makes it difficult to determine whether participants’ choice to prioritize one task over another was self-initiated or researcher-directed. This is especially important when a group differs in which task they are more likely to prioritize. For example, older adults and individuals with ID tend to prioritize balance performance over a concurrent non-balance task as a self-preserving strategy to maintain stability and prevent falls (Li et al., 2005; Yogev-Seligmann et al., 2012; Pineda et al., 2022).

Critical evidence for reduced dual-task ability in individuals with ID relative to controls is that group differences in dual-task performance is above and beyond group differences in single-task performance. This can be achieved by the use of proportional dual-task interference, which factors out individual differences in single-task performance and may be the preferred dual-task measure when single-task performance significantly differs between groups (Gagné et al., 2017). The downside is its poor reliability, which can be attributed to the resulting error inflation when systematic errors from measures of single- and dual-task performances are combined to calculate proportional dual-task interference (Yang et al., 2015). Unreliable measures can have serious consequences on research findings including decreased statistical power in detecting between-group (e.g., ID versus non-ID) or within-group (e.g., pre- versus post-intervention) differences and attenuated correlations between variables (e.g., correlation between dual-task performance and fall risk). Alternatively, instead of calculating proportional dual-task interference, single-task performance can be equated between groups to simplify detection of group differences in dual-task performance. The large discrepancy in intellectual functioning between individuals with and without ID, however, can make this challenging. Included studies in this review adopted several strategies to make single- and dual-task performance comparable such as matching by MA rather than CA (Piccirilli et al., 1991; Shaw, 1998; Van der Molen et al., 2007; Hocking et al., 2013) and individually calibrating cognitive load like the length of digit span (Van der Molen et al., 2007; Oka and Miura, 2008; Hocking et al., 2014) or difficulty of semantic category (Hocking et al., 2013, 2014). It is important to note that these strategies do not always successfully eliminate group differences in single-task performance (Oka and Miura, 2008). Some strategies may also be inappropriate for some tasks. For instance, the biomechanical differences in the performance of gait and balance tasks between children and adults make the use of MA-matched control for gait or balance dual-task studies problematic.

Performance decline was the most commonly reported pattern of dual-task effect in individuals with ID but several studies also showed apparent improvement in performance in dual-task conditions. Notably, enhanced performance in dual-task conditions was observed only in gait/balance dual-task studies. Task prioritization for safety’s sake justified this finding for one of the study (Pineda et al., 2022). However, this justification is difficult to rationalize for the three relevant studies that did not report performance on the concurrent task (Horvat et al., 2013; Hocking et al., 2014; Pena et al., 2019) but at least two possibilities can explain these findings. First, the *constrained action hypothesis* (Wulf et al., 2001; Huxhold et al., 2006) proposes that gait/balance performance benefits from the introduction of a concurrent task, which draws attention away from highly automatized gait/balance control processes. The alternative is to revisit what constitutes *improved* gait/balance performance. Speaking particularly about balance, reduced postural sway (as measured by center-of-pressure excursion) may be an adaptive response involving *freezing degrees of freedom* (Bernstein, 1967). This strategy simplifies the regulatory control of balance but results in postural stiffness, which is energy inefficient, less adaptable, and attention demanding (Stins et al., 2011; Pineda et al., 2022).

The more important question is whether individuals with ID have specific deficits in dual-tasking and the evidence is inconsistent on this matter. Most included studies in the review showed greater dual-task interferences in participants with ID relative to controls. However, a few studies reported otherwise and their methodological characteristics can help draw inferences on the nature of dual-task deficits in individuals with ID. First, the type of task and ID etiology of participants may influence whether disability-specific dual-task deficit can be observed because of cognitive profiles distinctive to some genetic syndromes (Kittler et al., 2008; Hocking et al., 2014). This underscores the importance of the ID sample’s composition in the interpretation of dual-task study findings. Second, concurrent tasks that are passive (e.g., listening to music) or involve no performance target (e.g., pedaling with no outcome measure) may have insufficient cognitive load to elicit ID-related dual-task interferences (Rao et al., 2017). Third, varying a task’s cognitive load according to cognitive abilities may eliminate differences in dual-task interference between individuals with ID and controls (Oka and Miura, 2008), suggesting a lack of ID-specific dual-task deficit. This is corroborated by Shaw’s (1998) study, which showed that individuals with ID had larger dual-task interferences compared to CA-controls but not MA-controls. In fact, the pattern of dual-task performance of MA-controls resembled that of individuals with ID. Fourth, group differences in dual-task interference may disappear if dual-task interferences for each single-tasks are averaged together into a single metric (Van der Molen et al., 2007). Although this may reflect a real absence of group difference in dual-task interference between individuals with ID and controls, conclusions based solely on the combined dual-task interference measure risk missing potentially divergent prioritization strategies between groups.

The current scoping review uncovered several gaps in the literature of dual-tasking in individuals with ID. Mapping out the included dual-task studies underscored the discordant methods used to measure dual-task performance. Reporting practices across the included studies were inconsistent, especially in terms of adequately describing the sample (both participants with ID and controls) and experimental tasks and procedures. This made it a challenge to interpret and compare findings between studies. Moreover, many studies have small sample sizes that may have been inadequately powered to address their respective research objectives, further adding to the difficulty in determining the effect of ID on dual-task interference. We also identified gaps in thematic content. For instance, only one study examined the effect of ID severity. Van Pelt et al. (2020) showed larger gait dual-task interferences in participants with borderline/mild relative to moderate ID, which they attribute to individual with moderate ID’s single-task gait velocity being slow enough that the concurrent task did not slow down gait further. The limited number of studies on dual-task training is another gap in the literature. Evidence from the included studies did not provide support for the trainability of dual-tasking. However, randomized controlled trials on older adults and individuals with neurological disorders have demonstrated the effectiveness of dual-task training. Better designed studies using randomized controlled trials are needed to determine whether dual-tasking ability can be trained in individuals with ID.

It is important to acknowledge the limitations of this scoping review. First, the search for relevant literature was limited to electronic databases available to the researchers and the search terms used, while relatively broad, did not cover all disorders that may present with ID (e.g., cerebral palsy and a number of genetic, metabolic and chromosomal disorders). Second, non-English language citations may be underrepresented in this review. Although we searched in non-English databases and included non-English citations, only articles with an English abstract were translated. Third, patterns of dual-task interference only covered groups’ average performance and did not incorporate performance variability because of inconsistent reporting. Examining the effect of dual-tasking on performance variability in ID is relevant given the well-known tendency toward larger performance variability in participants with ID relative to controls (Jenkinson, 1989; Lahtinen et al., 2007; Van Biesen et al., 2023). Finally, included studies were not appraised for quality, in alignment with scoping review methodology (Arksey and O’Malley, 2005; Levac et al., 2010). We describe in the next section suggested criteria for methodological quality assessment for dual-task studies, which can be used by prospective systematic reviews and meta-analyses.

### Implications and recommendations

The absence of methodological and reporting guidelines for dual-task studies hinders interpretation of dual-task findings and comparability between studies. To advance the application of dual-task paradigm for ID and other potential populations of interest, a “minimum criteria” for dual-task research is needed. These may include: (1) describe participants with ID and controls adequately (e.g., ID etiology, presence of comorbidities, IQ or other measures of intellectual functioning, matching criteria for controls); (2) measure dual-task interferences in all single-tasks; (3) equate single-task performance across groups; and (4) specify priority instruction. These are consistent with recommendations made by other researchers (Li et al., 2005; Schaefer, 2014; McIsaac et al., 2015; Plummer and Eskes, 2015).

The value of measuring dual-task interference extends beyond theoretical research applications. It has been proposed that taxing human information processing through the simultaneous performance of multiple tasks may have clinical use, especially in pathological conditions with cognitive deficits (Saccani et al., 2022). Two studies included in this review have tested the use of dual-task interference for a number of clinical applications, such as early Alzheimer detection in adults with DS (Van Pelt et al., 2020) and fall prediction in adults with ID (Oppewal and Hilgenkamp, 2019). Neither studies gave definitive conclusions to the diagnostic and prognostic value of dual-task performance, which is likely due to methodological limitations. Because dual-tasking is such an integral part of everyday life, researchers have acknowledged that dual-task conditions is the most valid context to identify performance difficulties in real life settings. In a similar vein, training in dual-task context is believed to simulate the conditions in which tasks are naturally performed and, thus, dual-task training can result to improvements that generalize to natural environments. For example, Mikolajczyk and Jankowicz-Szymanska (2015a,b) demonstrated that standing balance improved after dual-task training; however it is unclear if dual-task training is superior to other training or if dual-task training results to improvements in dual-task performance (rather than just improvement in balance). The clinical uses of dual-task testing are promising but until the methodological rigor and reporting standards of dual-task studies continue to be insufficient, these assumed potentials are unlikely to be fulfilled.

## Conclusion

This scoping review is the first to survey systematically the published literature on dual-task interference in individuals with ID. While the evidence is consistent regarding individuals with ID’s intact ability to dual-task, the pattern of dual-task interference is inconsistent. Owing to the varying dual-task procedures applied across studies, the evidence is inconclusive regarding dual-task-specific deficit in ID. To advance our understanding of the impact of ID on dual-task interference, researchers should be more cognisant of the methodological and interpretive issues of dual-task research in individuals with ID.

## Author contributions

RP developed the search strategy, ran the search in all the electronic databases, deduplicated records obtained from the database search, and wrote the initial draft of the manuscript. RP and DB screened the records, charted the data, and performed thematic analysis. RP, RK, YV, and DB were involved in the conception and design of the study. All authors have read, contributed to the revisions, and approved the submitted version of the manuscript.

## Funding

This work was supported by the Van Goethem-Brichant Foundation and the Flemish Fund for Scientific Research (FWO Grant no. G0C6817N). The funders had no role in study design, data collection and analysis, decision to publish, or preparation of the manuscript.

## Conflict of interest

The authors declare that the research was conducted in the absence of any commercial or financial relationships that could be construed as a potential conflict of interest.

## Publisher’s note

All claims expressed in this article are solely those of the authors and do not necessarily represent those of their affiliated organizations, or those of the publisher, the editors and the reviewers. Any product that may be evaluated in this article, or claim that may be made by its manufacturer, is not guaranteed or endorsed by the publisher.

## Supplementary material

SUPPLEMENTARY FIGURE S1 PRISMA flow diagram of the second iteration of study selection carried out in January 2022.
