# Supplementary material for: Scoping review of dual-task interference in individuals with intellectual disability
Source: Front Psychol. 2023 Aug 24;14:1223288. doi: 10.3389/fpsyg.2023.1223288 (PMC10484534; doi:10.3389/fpsyg.2023.1223288)
Supplement: Supplementary file 2 [file Table_2.pdf]

Supplemental Table 2 | Search strategy used for the various databases and study registers.

Database	Search string	Result Jan 2021	Jan 2022
Ovid MEDLINE / PubMed	(exp Intellectual Disability/ OR Learning Disabilities/ OR exp Mentally Disabled Persons/ OR (("intellectual*" OR "learning" OR "mental*") ADJ3 ("disab*" OR "impair*" OR "dysfunction*" OR "disorder*" OR "deficien*" OR "retard*" OR "handicap*" OR "challenged")).ti,ab,kf. OR ((("down" OR "downs" OR "down's") ADJ3 "syndrome*") OR ("trisom*" ADJ3 "21")).ti,ab,kf. OR ((("fragil*" OR "marker" OR "fra" OR "mar") ADJ3 "x") OR (("fraxa" OR "fraxe") ADJ3 "syndrome*") OR "martin bell").ti,ab,kf.) AND (exp Multitasking Behavior/ OR exp Executive Function/ OR ("dualtask*" OR "dual task*" OR "dual motor task*" OR "dual cognitive task*" OR "dual motor cognitive task*" OR "dual cognitive motor task*" OR "multitask*" OR "multi task*" OR "secondary task*" OR "concurrent task*" OR "simultaneous task*" OR "divided attention" OR "alternating attention" OR "task coordination" OR "task switching" OR "task interference").ti,ab,kf. OR ("executive function*" OR "executive control" OR "cognitive control" OR "central executive").ti,ab,kf.)	1,949	240
Embase	('intellectual impairment'/de OR 'mental deficiency'/exp OR 'learning disorder'/de OR 'mentally disabled person'/exp OR (('intellectual*' OR 'learning' OR 'mental*') NEAR/3 ('disab*' OR 'impair*' OR 'dysfunction*' OR 'disorder*' OR 'deficien*' OR 'retard*' OR 'handicap*' OR 'challenged')):ti,ab,kw OR (('down' OR 'downs' OR 'down s') NEAR/3 'syndrome*'):ti,ab,kw OR ('trisom*' NEAR/3 '21'):ti,ab,kw OR (('fragil*' OR 'marker' OR 'fra' OR 'mar') NEAR/3 'x'):ti,ab,kw OR (('fraxa' OR 'fraxe') NEAR/3 'syndrome*'):ti,ab,kw OR 'martin bell':ti,ab,kw) AND ('dual-task performance'/exp OR 'executive function'/exp OR 'dual task'/exp OR 'dualtask*':ti,ab,kw OR 'dual task*':ti,ab,kw OR 'dual motor task*':ti,ab,kw OR 'dual cognitive task*':ti,ab,kw OR 'dual motor cognitive task*':ti,ab,kw OR 'dual cognitive motor task*':ti,ab,kw OR 'multitask*':ti,ab,kw OR 'multi task*':ti,ab,kw OR 'secondary task*':ti,ab,kw OR 'concurrent task*':ti,ab,kw OR 'simultaneous task*':ti,ab,kw OR 'divided attention':ti,ab,kw OR 'alternating attention':ti,ab,kw OR 'task coordination':ti,ab,kw OR 'task switching':ti,ab,kw OR 'task interference':ti,ab,kw OR 'executive function*':ti,ab,kw OR 'executive control':ti,ab,kw OR 'cognitive control':ti,ab,kw OR 'central executive':ti,ab,kw)	3,618	417
Ovid PsycInfo†	(exp Intellectual Development Disorder/ OR learning disorders/ OR learning disabilities/ OR (("intellectual*" OR "learning" OR "mental*") ADJ3 ("disab*" OR "impair*" OR "dysfunction*" OR "disorder*" OR "deficien*" OR "retard*" OR "handicap*" OR "challenged")).ti,ab,id. OR ((("down" OR "downs" OR "down's") ADJ3 "syndrome*") OR ("trisom*" ADJ3 "21")). ti,ab,id. OR ((("fragil*" OR "marker" OR "fra" OR "mar") ADJ3 "x") OR (("fraxa" OR "fraxe") ADJ3 "syndrome*") OR "martin bell").ti,ab,id.) AND (exp Executive Function/ OR exp dual task performance/ OR exp multitasking/ OR ("dualtask*" OR "dual task*" OR "dual motor task*" OR "dual cognitive task*" OR "dual motor cognitive task*" OR "dual cognitive motor task*" OR "multitask*" OR "multi task*" OR "secondary task*" OR "concurrent task*" OR "simultaneous task*" OR "divided attention" OR "alternating attention" OR "task coordination" OR "task switching" OR "task interference").ti,ab,id. OR ("executive function*" OR "executive control" OR "cognitive control" OR "central executive").ti,ab,id.)	1,876	140

Ovid ERIC (Education Resources Information Center)	exp intellectual disability/ OR exp learning disabilities/ OR (("intellectual*" OR "learning" OR "mental*") ADJ3 ("disab*" OR "impair*" OR "dysfunction*" OR "disorder*" OR "deficien*" OR "retard*" OR "handicap*" OR "challenged")).ti,ab,id. OR ((("down" OR "downs" OR "down's") ADJ3 "syndrome*") OR ("trisom*" ADJ3 "21")). ti,ab,id. OR ((("fragil*" OR "marker" OR "fra" OR "mar") ADJ3 "x") OR (("fraxa" OR "fraxe") ADJ3 "syndrome*") OR "martin bell").ti,ab,id.) AND (exp executive function/ OR ("dualtask*" OR "dual task*" OR "dual motor task*" OR "dual cognitive task*" OR "dual motor cognitive task*" OR "dual cognitive motor task*" OR "multitask*" OR "multi task*" OR "secondary task*" OR "concurrent task*" OR "simultaneous task*" OR "divided attention" OR "alternating attention" OR "task coordination" OR "task switching" OR "task interference").ti,ab,id. OR ("executive function*" OR "executive control" OR "cognitive control" OR "central executive").ti,ab,id.)	315	2
EBSCO CINAHL (Cumulative Index to Nursing and Allied Health Literature)	((MH "Intellectual Disability+") OR (MH "Mentally Disabled Persons") OR (TI((intellectual* OR learning OR mental*) N3 (disab* OR impair* OR dysfunction* OR disorder* OR deficien* OR retard* OR handicap* OR challenged)) OR AB ((intellectual* OR learning OR mental*) N3 (disab* OR impair* OR dysfunction* OR disorder* OR deficien* OR retard* OR handicap* OR challenged))) OR (TI(((down OR downs OR "down's") N3 syndrome*) OR (trisom* N3 21)) OR AB(((down OR downs OR "down's") N3 syndrome*) OR (trisom* N3 21))) OR (TI(((fragil* OR marker OR fra OR mar) N3 x) OR ((fraxa OR fraxe) N3 syndrome*) OR "martin bell")) OR AB(((fragil* OR marker OR fra OR mar) N3 x) OR ((fraxa OR fraxe) N3 syndrome*) OR "martin bell"))) AND ((MH "Executive Function") OR (MH "Multitasking Behavior") OR (TI(dualtask* OR "dual task*" OR "dual motor task*" OR "dual cognitive task*" OR "dual motor cognitive task*" OR "dual cognitive motor task*" OR multitask* OR "multi task*" OR "secondary task*" OR "concurrent task*" OR "simultaneous task*" OR "divided attention" OR "alternating attention" OR "task coordination" OR "task switching" OR "task interference") OR AB(dualtask* OR "dual task*" OR "dual motor task*" OR "dual cognitive task*" OR "dual motor cognitive task*" OR "dual cognitive motor task*" OR multitask* OR "multi task*" OR "secondary task*" OR "concurrent task*" OR "simultaneous task*" OR "divided attention" OR "alternating attention" OR "task coordination" OR "task switching" OR "task interference")) OR (TI("executive function*" OR "executive control" OR "cognitive control" OR "central executive") OR AB("executive function*" OR "executive control" OR "cognitive control" OR "central executive"))	646	73
EBSCO SPORTDiscus	((DE "MENTAL disabilities") OR (DE "DOWN syndrome") OR (DE "PRADER-Willi syndrome") OR (ZU "fragile x syndrome") OR (DE "PEOPLE with mental disabilities") OR (DE "PHYSICAL education for people with mental disabilities") OR (DE "PHYSICAL education for children with mental disabilities") OR (DE "PHYSICAL education for youth with mental disabilities") OR (ZU "mental retardation") OR (ZU "learning disabled children") OR (ZU "learning disabled persons") OR (ZU "learning disabled teenagers") OR (ZU "learning disabled women") OR (ZU "learning disabled youth") OR (ZU "learning disabilities") OR (ZU "learning disabilities research") OR (TI((intellectual* OR learning OR mental*) N3 (disab* OR impair* OR dysfunction* OR disorder* OR deficien* OR retard* OR handicap* OR challenged)) OR AB((intellectual* OR learning OR mental*) N3 (disab* OR impair* OR dysfunction* OR	71	6

	disorder* OR deficien* OR retard* OR handicap* OR challenged)) OR KW((intellectual* OR learning OR mental*) N3 (disab* OR impair* OR dysfunction* OR disorder* OR deficien* OR retard* OR handicap* OR challenged))) OR (TI(((down OR downs OR "down’s") N3 syndrome*) OR (trisom* N3 21)) OR AB(((down OR downs OR "down’s") N3 syndrome*) OR (trisom* N3 21)) OR KW(((down OR downs OR "down’s") N3 syndrome*) OR (trisom* N3 21))) OR (TI(((fragil* OR marker OR fra OR mar) N3 x) OR ((fraxa OR fraxe) N3 syndrome*) OR "martin bell")) OR AB(((fragil* OR marker OR fra OR mar) N3 x) OR ((fraxa OR fraxe) N3 syndrome*) OR "martin bell")) OR KW(((fragil* OR marker OR fra OR mar) N3 x) OR ((fraxa OR fraxe) N3 syndrome*) OR "martin bell"))) AND ((ZU "executive function (neuropsychology)") OR (TI(dualtask* OR "dual task*" OR "dual motor task*" OR "dual cognitive task*" OR "dual motor cognitive task*" OR "dual cognitive motor task*" OR multitask* OR "multi task*" OR "secondary task*" OR "concurrent task*" OR "simultaneous task*" OR "divided attention" OR "alternating attention" OR "task coordination" OR "task switching" OR "task interference") OR AB(dualtask* OR "dual task*" OR "dual motor task*" OR "dual cognitive task*" OR "dual motor cognitive task*" OR "dual cognitive motor task*" OR multitask* OR "multi task*" OR "secondary task*" OR "concurrent task*" OR "simultaneous task*" OR "divided attention" OR "alternating attention" OR "task coordination" OR "task switching" OR "task interference") OR KW(dualtask* OR "dual task*" OR "dual motor task*" OR "dual cognitive task*" OR "dual motor cognitive task*" OR "dual cognitive motor task*" OR multitask* OR "multi task*" OR "secondary task*" OR "concurrent task*" OR "simultaneous task*" OR "divided attention" OR "alternating attention" OR "task coordination" OR "task switching" OR "task interference")) OR (TI("executive function*" OR "executive control" OR "cognitive control" OR "central executive") OR AB("executive function*" OR "executive control" OR "cognitive control" OR "central executive") OR KW("executive function*" OR "executive control" OR "cognitive control" OR "central executive"))		
Web of Science Core Collection	(TS=((intellectual* OR "learning" OR mental*) NEAR/3 (disab* OR impair* OR dysfunction* OR disorder* OR deficien* OR retard* OR handicap* OR "challenged")) OR TS=((("down" OR "downs" OR "down s") NEAR/3 syndrome*) OR (trisom* NEAR/3 "21")) OR TS=(((fragil* OR "marker" OR "fra" OR "mar") NEAR/3 "x") OR (("fraxa" OR "fraxe") NEAR/3 syndrome*) OR "martin bell")) AND (TS=("dual task*" OR dualtask* OR "dual motor task*" OR "dual cognitive task*" OR "dual motor cognitive task*" OR "dual cognitive motor task*" OR "multi task*" OR multitask* OR "secondary task*" OR "concurrent task*" OR "simultaneous task*" OR "divided attention" OR "alternating attention" OR "task coordination" OR "task switching" OR "task interference") OR TS=("executive function*" OR "executive control" OR "cognitive control" OR "central executive"))	2,814	315
Web of Science SciELO Citation Index	(TS=((intellectual* OR "learning" OR mental*) NEAR/3 (disab* OR impair* OR dysfunction* OR disorder* OR deficien* OR retard* OR handicap* OR "challenged")) OR TS=((("down" OR "downs" OR "down s") NEAR/3 syndrome*) OR (trisom* NEAR/3 "21")) OR TS=(((fragil* OR "marker" OR "fra" OR "mar") NEAR/3 "x") OR (("fraxa" OR "fraxe") NEAR/3 syndrome*) OR "martin bell")) AND (TS=("dual task*" OR dualtask* OR "dual motor task*" OR "dual cognitive task*" OR "dual motor cognitive task*" OR "dual cognitive motor task*" OR	41	0

	"multi task*" OR multitask* OR "secondary task*" OR "concurrent task*" OR "simultaneous task*" OR "divided attention" OR "alternating attention" OR "task coordination" OR "task switching" OR "task interference") OR TS=("executive function*" OR "executive control" OR "cognitive control" OR "central executive"))		
Web of Science Chinese Science Citation Database	TS=((intellectual* OR "learning" OR mental*) NEAR/3 (disab* OR impair* OR dysfunction* OR disorder* OR deficien* OR retard* OR handicap* OR "challenged")) OR TS=((("down" OR "downs" OR "down s") NEAR/3 syndrome*) OR (trisom* NEAR/3 "21")) OR TS=(((fragil* OR "marker" OR "fra" OR "mar") NEAR/3 "x") OR (("fraxa" OR "fraxe") NEAR/3 syndrome*) OR "martin bell")) AND (TS=("dual task*" OR dualtask* OR "dual motor task*" OR "dual cognitive task*" OR "dual motor cognitive task*" OR "dual cognitive motor task*" OR "multi task*" OR multitask* OR "secondary task*" OR "concurrent task*" OR "simultaneous task*" OR "divided attention" OR "alternating attention" OR "task coordination" OR "task switching" OR "task interference") OR TS=("executive function*" OR "executive control" OR "cognitive control" OR "central executive"))	80	9
Web of Science KCI-Korean Journal Database	(TS=((intellectual* OR "learning" OR mental*) NEAR/3 (disab* OR impair* OR dysfunction* OR disorder* OR deficien* OR retard* OR handicap* OR "challenged")) OR TS=((("down" OR "downs" OR "down s") NEAR/3 syndrome*) OR (trisom* NEAR/3 "21")) OR TS=(((fragil* OR "marker" OR "fra" OR "mar") NEAR/3 "x") OR (("fraxa" OR "fraxe") NEAR/3 syndrome*) OR "martin bell")) AND (TS=("dual task*" OR dualtask* OR "dual motor task*" OR "dual cognitive task*" OR "dual motor cognitive task*" OR "dual cognitive motor task*" OR "multi task*" OR multitask* OR "secondary task*" OR "concurrent task*" OR "simultaneous task*" OR "divided attention" OR "alternating attention" OR "task coordination" OR "task switching" OR "task interference") OR TS=("executive function*" OR "executive control" OR "cognitive control" OR "central executive"))	34	5
Web of Science Russian Science Citation Index‡	(TS=((intellectual* OR "learning" OR mental*) NEAR/3 (disab* OR impair* OR dysfunction* OR disorder* OR deficien* OR retard* OR handicap* OR "challenged")) OR TS=((("down" OR "downs" OR "down s") NEAR/3 syndrome*) OR (trisom* NEAR/3 "21")) OR TS=(((fragil* OR "marker" OR "fra" OR "mar") NEAR/3 "x") OR (("fraxa" OR "fraxe") NEAR/3 syndrome*) OR "martin bell")) AND (TS=("dual task*" OR dualtask* OR "dual motor task*" OR "dual cognitive task*" OR "dual motor cognitive task*" OR "dual cognitive motor task*" OR "multi task*" OR multitask* OR "secondary task*" OR "concurrent task*" OR "simultaneous task*" OR "divided attention" OR "alternating attention" OR "task coordination" OR "task switching" OR "task interference") OR TS=("executive function*" OR "executive control" OR "cognitive control" OR "central executive"))	12	7
Scopus	(TITLE-ABS-KEY((intellectual* OR learning OR mental*) W/3 (disab* OR impair* OR dysfunction* OR disorder* OR deficien* OR retard* OR handicap* OR challenged)) OR TITLE-ABS-KEY(((down OR downs) W/3 syndrome*) OR (trisom* W/3 21)) OR TITLE-ABS-KEY(((fragil* OR marker OR fra OR mar) W/3 x) OR ((fraxa OR fraxe) W/3 syndrome*) OR "martin bell")) AND (TITLE-ABS-KEY(dualtask* OR "dual task*" OR "dual motor task*" OR "dual cognitive task*" OR "dual motor cognitive task*" OR "dual cognitive motor task*" OR multitask* OR "multi	4,432	436

	task*" OR "secondary task*" OR "concurrent task*" OR "simultaneous task*" OR "divided attention" OR "alternating attention" OR "task coordination" OR "task switching" OR "task interference") OR TITLE-ABS-KEY("executive function*" OR "executive control" OR "cognitive control" OR "central executive"))		
Proquest PsycArticles	(MAINSUBJECT.EXACT.EXPLODE("Intellectual Development Disorder") OR MAINSUBJECT.EXACT("Learning Disorders") OR MAINSUBJECT.EXACT("Learning Disabilities") OR MAINSUBJECT.EXACT("Fragile X Syndrome") OR TI,AB,IF((intellectual* OR learning OR mental*) NEAR/3 (disab* OR impair* OR dysfunction* OR disorder* OR deficien* OR retard* OR handicap* OR challenged)) OR TI,AB,IF(((down OR downs OR "down s") NEAR/3 syndrome*) OR (trisom* NEAR/3 21)) OR TI,AB,IF(((fragil* OR marker OR fra OR mar) NEAR/3 x) OR ((fraxa OR fraxe) NEAR/3 syndrome*) OR "martin bell") AND (MAINSUBJECT.EXACT.EXPLODE("Multitasking") OR MAINSUBJECT.EXACT.EXPLODE("Dual Task Performance") OR MAINSUBJECT.EXACT.EXPLODE("Executive Function") OR TI,AB,IF(dualtask* OR "dual task*" OR "dual motor task*" OR "dual cognitive task*" OR "dual motor cognitive task*" OR "dual cognitive motor task*" OR multitask* OR "multi task*" OR "secondary task*" OR "concurrent task*" OR "simultaneous task*" OR "divided attention" OR "alternating attention" OR "task coordination" OR "task switching" OR "task interference") OR TI,AB,IF("executive function*" OR "executive control" OR "cognitive control" OR "central executive"))	76	5
Proquest Central	(MAINSUBJECT.EXACT("Intellectual disabilities") OR MAINSUBJECT.EXACT("Learning disabilities") OR MAINSUBJECT.EXACT("Down syndrome") OR TI,AB,IF((intellectual* OR learning OR mental*) NEAR/3 (disab* OR impair* OR dysfunction* OR disorder* OR deficien* OR retard* OR handicap* OR challenged)) OR TI,AB,IF(((down OR downs OR "down’s") NEAR/3 syndrome*) OR (trisom* NEAR/3 21)) OR TI,AB,IF(((fragil* OR marker OR fra OR mar) NEAR/3 x) OR ((fraxa OR fraxe) NEAR/3 syndrome*) OR "martin bell")) AND (MAINSUBJECT.EXACT("Multitasking") OR MAINSUBJECT.EXACT("Executive function") OR TI,AB,IF(dualtask* OR "dual task*" OR "dual motor task*" OR "dual cognitive task*" OR "dual motor cognitive task*" OR "dual cognitive motor task*" OR multitask* OR "multi task*" OR "secondary task*" OR "concurrent task*" OR "simultaneous task*" OR "divided attention" OR "alternating attention" OR "task coordination" OR "task switching" OR "task interference") OR TI,AB,IF("executive function*" OR "executive control" OR "cognitive control" OR "central executive"))	1,118	161
Cochrane Central	([mh "Intellectual Disability"] OR [mh "Mentally Disabled Persons"] OR [mh ^"Learning Disabilities"] OR ((intellectual* OR "learning" OR "mental*") NEAR/3 (disab* OR impair* OR dysfunction* OR disorder* OR deficien* OR retard* OR handicap* OR "challenged")):ti,ab,kw OR (((down OR downs OR "down’s") NEAR/3 syndrome*) OR (trisom* NEAR/3 21)):ti,ab,kw OR (((fragil* OR marker OR fra OR mar) NEAR/3 x) OR ((fraxa OR fraxe) NEAR/3 syndrome*) OR "martin bell"):ti,ab,kw) AND ([mh "Multitasking Behavior"] OR [mh "Executive Function"] OR (dualtask* OR (dual NEXT task*) OR (dual NEXT (motor OR cognitive) NEXT task*) OR ("dual motor cognitive" NEXT task*) OR ("dual cognitive motor" NEXT task*) OR multitask* OR (multi NEXT task*) OR (secondary NEXT	462	59

	task*) OR (concurrent NEXT task*) OR (simultaneous NEXT task*) OR "divided attention" OR "alternating attention" OR "task coordination" OR "task switching" OR "task interference"):ti,ab,kw OR ((executive NEXT function*) OR "executive control" OR "cognitive control" OR "central executive"):ti,ab,kw)		
ClinicalTrials.gov	Condition or Disease: Intellectual disability<br>Study type: -<br>Study results: Studies with Results	71	12
International Clinical Trials Registry Platform	intellectual disabilities OR mental retardation OR learning disabilities OR developmental disorders	158	42

†Database was searched through Proquest in the second iteration of the search due to a change in access provided by the researcher's university library
