# Supplementary figures and images for: Scoping review of dual-task interference in individuals with intellectual disability
Source: Front Psychol. 2023 Aug 24;14:1223288. doi: 10.3389/fpsyg.2023.1223288 (PMC10484534; doi:10.3389/fpsyg.2023.1223288)

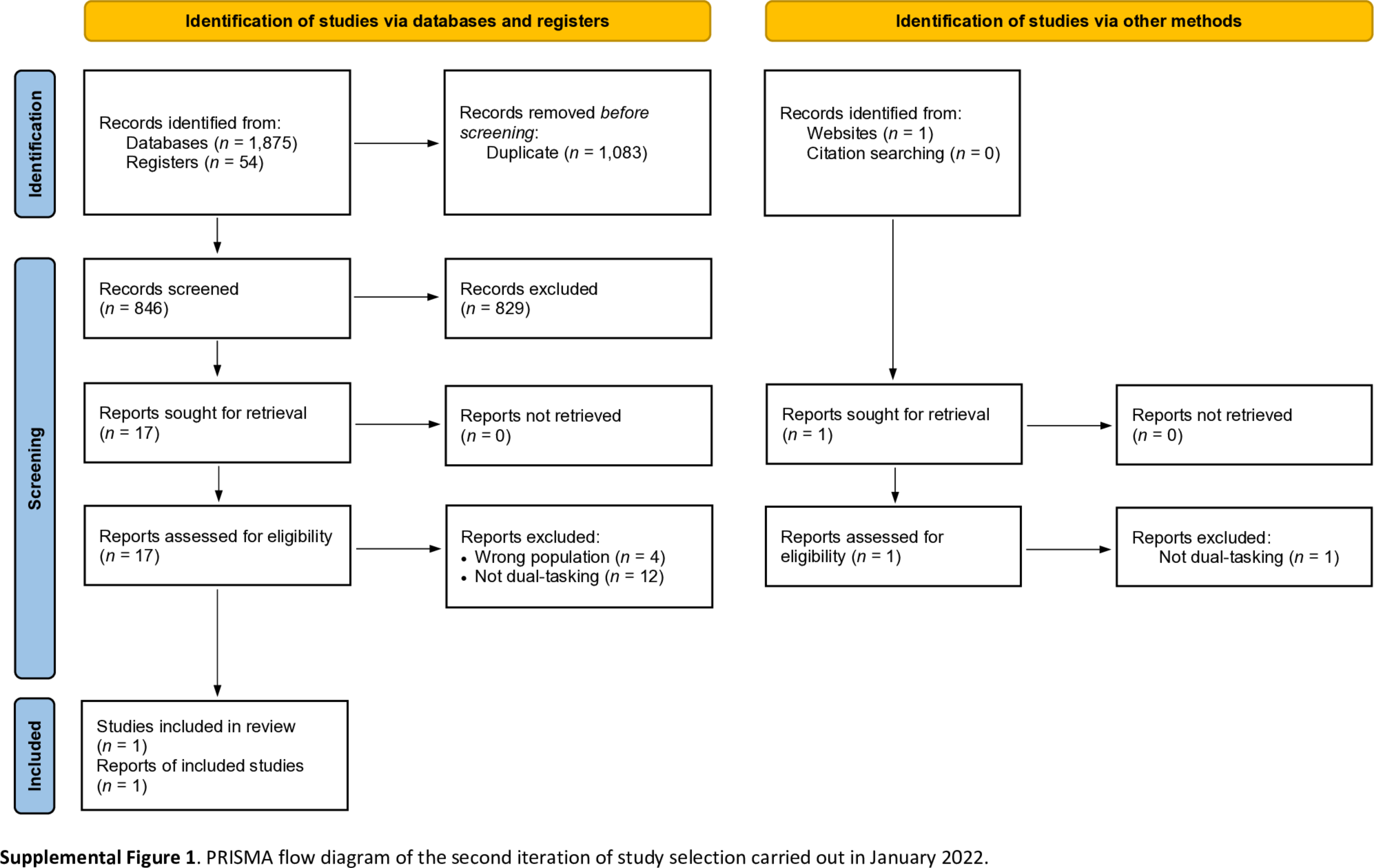

Supplement: SUPPLEMENTARY FIGURE S1 — PRISMA flow diagram of the second iteration of study selection carried out in January 2022. [file Image_1.TIFF]
